# Tendoscopic versus open release for de Quervain’s disease: earlier recovery with 7.21 year follow-up

**DOI:** 10.1186/s13018-019-1393-5

**Published:** 2019-11-12

**Authors:** Xiao-hui Gu, Zhe-ping Hong, Xin-ji Chen, Yu Tong, Jian-fei Hong, Zong-ping Luo, Qing Bi

**Affiliations:** 1 Orthopaedic Institute, Department of Orthopaedics, The First Affiliated Hospital of SooChow University, 708 Renmin Rd, Suzhou, 215007 Jiangsu China; 2 Department of Orthopaedic Surgery, Zhejiang Provincial People’s Hospital and People’s Hospital of Hangzhou Medical College, No. 158 Shangtang Road, Hangzhou, 310014 Zhejiang China; 3 The Second Affiliated Hospital of Wenzhou Medical University, Wenzhou City, Zhejiang Province China

## Abstract

**Purpose:**

To compare the time return to work and long-term results of tendoscopic versus open technique for de Quervain’s disease.

**Methods:**

From 2005 to 2013, either tendoscopic or open decompression was performed on 56 consecutive patients (56 wrists) with symptomatic de Quervain’s disease despite a minimum of 3 months non-operative treatment. Of the 50 patients who met the inclusion criteria, 41 patients were followed-up for a mean of 7.21 years postoperatively. Among these 41 wrists, 20 underwent tendoscopic release (group A), and 21 underwent open release (group B). The clinical evaluations were performed preoperatively, 1 month postoperatively and at last follow-up visit, using visual analog scale (VAS); the Disabilities of the Arm, Shoulder and Hand (DASH) Outcome score; and the Finkelstein’s test. The Patient and Observer Scar Assessment Scale (POSAS) was used as an esthetic evaluation tool of the scar at last follow-up.

**Results:**

No significant baseline differences were found between two groups. The average time return to work in group A was less than in group B (*P* < 0.05), The mean VAS and DASH scores improved significantly in both groups at 1 month and last follow-up visit (*P* < 0.001). At 1 month, the scores in group A were significantly better than in group B (*P* < 0.05 and *P* < 0.001, respectively). There was no difference between groups at last follow-up. In addition, the improvement of the mean DASH score was significantly greater in group A than in group B (34.74 ± 10.99 in group A and 23.58 ± 12.01 in group B, *P* < 0.01) at 1 month. For POSAS scale, both the OSAS and PSAS scores were significantly better in group A. One patient in group A had cephalic vein injury and 3 patients in group B was involved with radial sensory nerve injury. All patients showed negative on Finkelstein’s test at last follow-up.

**Conclusions:**

The results of this study suggest that tendoscopic technique for de Quervain’s disease could provide earlier symptom relief and earlier recovery with fewer complications and more desirable scar, as well as equivalent successful long-term outcome, when compared with traditional open release technique.

## Introduction

De Quervain’s disease, or stenosing tenosynovitis of the first dorsal compartment of the radial styloid process, is a common affliction with a prevalence of 0.5% among men and 1.3% among women [1]. The cause of this disease is thought to be the thickening of the tendon sheath containing the abductor pollicis longus (APL) and the extensor pollicis brevis (EPB) tendons [2]. Nonsurgical management, including rest, splint immobilization, physiotherapy, and steroid injections into the tendon sheath, could provide effective symptoms relief in most of the patients [3–5]. In resistant cases, surgical release of the first dorsal compartment is recommended. Open release has been widely studied since Fritz de Quervain firstly published the surgical technique in an article in 1895 [6]. Endoscopic release for de Quervain’s disease was firstly described by Slade and Merrell in 2007 [7] and was then studied by several researches for its good results and fewer complications [8–10]. We prefer to call it tendoscopic rather than endoscopic as the procedure is done around the APL and EPB tendons, and to our knowledge none of them reported the long-term results of this minimum-invasive technique. We presented our study here to compare the time return to work and long-term results of tendoscopic technique with open release for de Quervain’s disease.

## Materials and methods

We retrospectively reviewed 56 consecutive patients (56 wrists) with a diagnosis of de Quervain’s disease who were treated with tendoscopic or open release from March 2005 to June 2013. All patients showed pain and tenderness over the first dorsal compartment and a positive sign of Finkelstein test despite a minimum of 3 months non-operative treatment, including rest, splinting, and steroid injection (0.5 ml betamethasone, DIPROSPAN®, MSD and 1 ml of 1% lidocaine) for up to three courses. Patients were excluded if: (1) patients had secondary arthritis to the wrist (related to rheumatoid arthritis, gouty arthritis, or post-infectious arthritis), (2) patients had previous traumatic injury to the wrist, (3) patients had bone hyperplasia at the radial styloid, and (4) patients were involved with other symptomatic upper limb disorders, such as adhesive capsulitis, rotator cuff tear, lateral epicondylitis, and carpal tunnel syndrome etc. Of a total of 56 patients, 50 patients met the study inclusion criteria; 41 (82%) were available for follow-up assessment. These 41 patients were divided into two groups: 20 patients who were treated with tendoscopic release (group A); 21 patients who were handled with open release (group B).

### Surgical technique

In the pre-operative preparation, patients were instructed to extend their thumb, and a rubber compression cord was tied up in the proximal side of the upper limb to engorge the cephalic vein. The course of the vein was then marked around the radial styloid. The radial sensory nerve was located and marked by percussion around the radial styloid. After checking the patient information by the operator, anesthetist, and circulating nurse, the operations were performed under tourniquet control with brachial plexus anesthesia.

In the open release group, a 3-cm-length longitudinal incision was made over the first extensor compartment of the radial styloid process. The radial sensory nerve and the cephalic vein were meticulously identified and protected by blunt decollement of the subcutaneous tissues. The first compartment was then exposed and the extensor retinaculum was opened at its most ulnar corner. If there was a supernumerary septum it was also resected. By abducing the thumb, the decompression of the tendons was identified. The tourniquet was then released and adequate hemostasis completed. The incision was closed with a 4–0 subcuticular suture and a dressing was applied.

**Fig. 1:**
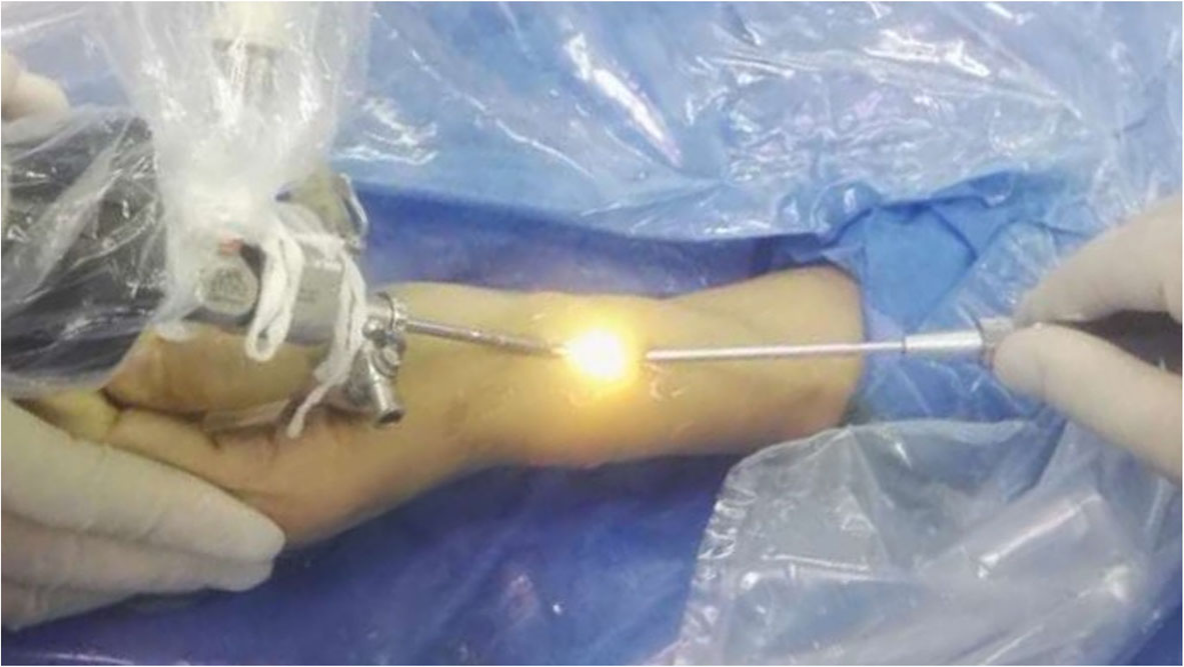
Our surgical portals: one 1.5 cm distal and the other 2–2.5 cm proximal to the radial styloid

**Fig. 2:**
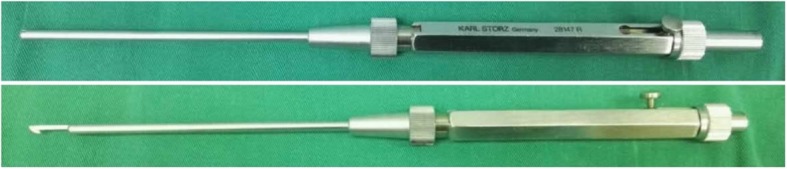
The telescopic knife (KARL STORZ, Germany) with the blade inside (above) and outside (below)

**Fig. 3:**
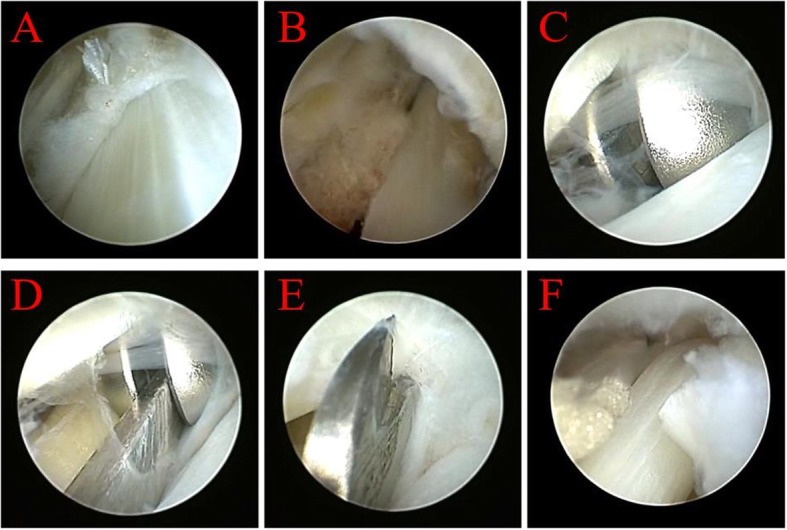
Endoscopic view: **a** the extensor retinaculum and the compressed tendons; **b** the inflammatory synovial hyperplasia inside; **c** the blade remained retracting inside of the blunt tip; **d** the blade was extended out of the knife; **e** endoscopic release with the blade; **f** the extensor retinaculum was opened at its ulnar side

In the tendoscopic release group, two 2-mm transverse portals were made: one 1.5 cm distal and the other 2–2.5 cm proximal to the radial styloid (Fig. 1). Two portals were alternatively used for visualization or manipulation in case of a variation in the anatomy of the first dorsal compartment. The radial sensory nerve and the cephalic vein were carefully protected. At the distal portal, blunt dissection exposed the EPB and APL tendon just distal to the first extensor compartment. A 2.7-mm, 30° arthroscope (KARL STORZ, Germany) was then introduced through the distal portal. After debridement of the synovitis, the first extensor compartment containing EPB and APL tendons could be seen. Then, a telescopic knife (KARL STORZ, Germany) (Fig. 2) was introduced through the proximal portal with care to avoid neurovascular and tendon injuries. The blade of this knife was not extended out of its blunt tip until it was placed in an eligible position that the first extensor compartment could be safely opened at its ulnar side. Any supernumerary septum seen tendoscopically was also resected (Fig. 3). Complete decompression of EPB and APL tendons was reconfirmed by abducing the thumb and elevating the tendons with a probe. The skin was closed using sterile infusion fixation pasters and a compressive dressing was applied.

Postoperatively, patients in both groups were encouraged to move their thumb and wrist with no immobilization.

### Assessment of clinical outcomes

The operative time, hospital costs, and time return to work in two groups were compared. Clinical results were assessed with use of the visual analog scale (VAS) for pain; the Disabilities of the Arm, Shoulder and Hand (DASH) Outcome score; and the Finkelstein’s test preoperatively, 1 month postoperatively and at the last follow-up visit. The Patient and Observer Scar Assessment Scale (POSAS) [11, 12] was used as an esthetic evaluation tool of the scar at last follow-up. The POSAS include the Observer Scar Assessment Scale (OSAS) (score range 5 to 50☹) and the Patient Scar Assessment Scale (PSAS) (score range 6 to 60☹). The POSAS is the only scar evaluation tool that comprises both the patient’s subjective opinion and observer’s assessment.

### Statistical analysis

All data are presented as means ± SD. We used SPSS software (version 20.0, IBM Corporation, NY, USA) for all data calculation. Differences between the groups were compared by using Pearson’s chi-squared test or Continuity Correction chi-squared test for categorical variables and the Student’s *t* test for continuous variables. Differences with *P* < 0.05 were considered statistically significant.

## Results

**Fig. 4:**
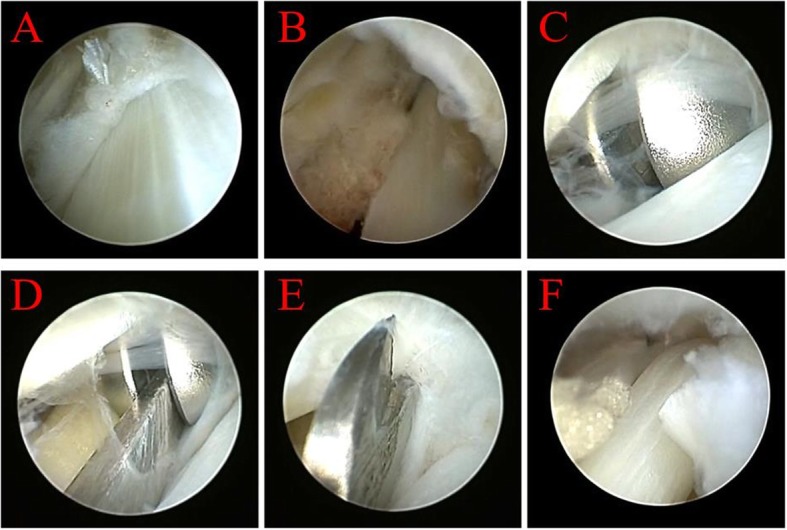
The VAS and DASH score preoperatively, 1 month postoperatively, and at the last follow-up visit. Values in graphs are expressed as mean ± SE in vertical bars, ***P < 0.001, statistical difference between two follow-up points

No significant difference of basic characteristics was found between groups regarding gender, age, profession, pre-operative duration of symptoms, pre-operative pain and disability scores or follow-up period (Table 1). The average operative time was 19.62 ± 4.17 min in group A and 17.87 ± 3.42 min in group B (*P* = 0.324). The average time return to work was 5.1 ± 1.37 days in group A and 15.33 ± 4.89 days in group B (*P* = 0.006). The average hospital cost was $362.00 ± 20.30 in group A and $429.38 ± 25.85 in group B (*P* = 0.137). Clinical outcomes were shown in Table 2 and Fig. 4. The mean VAS and DASH scores decreased significantly in both groups at 1 month and last follow-up visit (*P* < 0.001). At one month, the mean VAS score and DASH score in group A were significantly better than in group B (*P* < 0.05 and *P* < 0.001, respectively) (Table 2). There was no difference between groups at last follow-up. However, changes of the mean VAS pain score were not significantly different between groups at neither 1 month postoperative (*p* = 0.062) nor last follow-up visit (*p* = 0.862), compared with baseline. In addition, the improvement of the mean DASH score in group A was significantly greater at 1 month than in group B (34.74 ± 10.99 in group A and 23.58 ± 12.01 in group B, *P* < 0.01). However, the changes from baseline to last follow-up visit showed no significant difference between two groups (52.36 ± 11.87 in group A and 49.25 ± 14.55 in group B, *P* = 0.460). The surgical scar was evaluated using the POSAS scale, as shown in Table 2. Both the OSAS and PSAS scores were significantly better at last follow-up point in the tendoscopic release group.

**Table 1:** Comparison of baseline characteristics of two groups

	Group A (*n* = 20)	Group B (*n* = 21)	*P* value
Age (years)	48 ± 10.59	49 ± 8.61	0.856
Gender (male/female)	5/15	7/14	0.558
Dominant hand involvement (*n*, %)	11 (55.00)	10 (47.62)	0.636
High wrist demand profession (*n*, %)	16 (80.00)	15 (71.43)	0.783
Pre-op duration (months)	12.75 ± 6.05	10.57 ± 5.13	0.220
Follow-up period (years)	6.75 ± 1.86	7.67 ± 2.18	0.156
Baseline VAS score	7.00 ± 1.30	6.86 ± 1.31	0.728
Baseline DASH score	57.15 ± 14.19	55.40 ± 14.73	0.700

Values are expressed as mean ± SD unless otherwise indicated

*Abbreviation*: *VAS* visual analog scale, *DASH* Disabilities of the Arm, Shoulder and Hand scale

**Table 2:** Clinical outcomes of two groups

	Group A (*n* = 20)	Group B (*n* = 21)	*P* value
VAS score			
Pre-op	7.00 ± 1.30	6.86 ± 1.31	0.728
Δ. Pre-1 month	4.75 ± 1.48	3.90 ± 1.34	0.062
One month post-op	2.25 ± 1.07	2.95 ± 0.67	< 0.05
Δ. Pre-last FU	6.45 ± 1.28	6.38 ± 1.24	0.862
Last follow-up	0.55 ± 0.51	0.48 ± 0.68	0.697
DASH score			
Pre-op	57.15 ± 14.19	55.40 ± 14.73	0.700
Δ. Pre-1 month	34.74 ± 10.99	23.58 ± 12.01	< 0.01
One month post-op	22.42 ± 5.86	31.82 ± 8.28	< 0.001
Δ. Pre-last FU	52.36 ± 11.87	49.25 ± 14.55	0.460
Last follow-up	4.80 ± 4.06	6.14 ± 3.63	0.269
POSAS score at last follow-up			
OSAS	7.70 ± 1.26	8.20 ± 1.85	< 0.001
PSAS	12.38 ± 3.04	13.67 ± 3.37	< 0.001
Complications			
1. Radial sensory nerve injury	0	3	
2. Cephalic vein injury	1	0	

Values are expressed as mean ± SD unless otherwise indicated

*Abbreviation*: *VAS* visual analog scale, *FU* follow-up, *DASH* Disabilities of the Arm, Shoulder and Hand scale, *POSAS* the Patient and Observer Scar Assessment Scale, *OSAS* the Observer Scar Assessment Scale, *PASA* the Patient Scar Assessment Scale

One patient in group A had cephalic vein injury and finally got relieved by manual compression. Three patients in group B were involved with radial sensory nerve injury but had no signs of neuroma. No patients had tendon subluxation or symptom relapse. In addition, all patients showed negative on Finkelstein’s test at last follow-up visit.

## Discussion

To our knowledge, conservative treatment could effectively relieve pain and improve joint motion in acute phase of de Quervain’s disease. However, its long-term efficiency is dissatisfied with high recurrence rate. Surgical treatment is indicated following the failure of non-operative treatment for symptomatic de Quervain’s disease. Complications associated with surgical release include injury to cephalic vein or the radial sensory nerve, tendon subluxation postoperatively, hypertrophic and/or painful scarring, wound infection or delayed wound healing, and shoulder hand syndrome [13–19]. Iatrogenic injury to the radial sensory nerve is a common complication following the decompression [17]. Therefore, searching for a minimally invasive surgery procedure is of great value for those intractable de Quervain’s disease.

In this retrospective study, we reported our findings comparing open versus tendoscopic release for 41 patients (41 wrists) with a mean follow-up period of 7.21 years (range, 4–12 years). The average time return to work was significantly less in group A than group B, benefiting from less invasive procedure and earlier symptom relief. Our data demonstrated that tendoscopic release provided better clinical outcomes in terms of VAS and DASH scale at 1 month follow-up. In Kang’s randomized trial [10], short-term advantages of tendoscopic release was also confirmed that the clinical outcomes were significantly better in tendoscopic release group at 1 month follow-up. In Kang’s randomized trial [10], short-term advantages of tendoscopic release was also confirmed that the clinical outcomes were significantly better in tendoscopic release group at 12 weeks, but there was no significant difference between tendoscopic and open release at 24 weeks. Our follow-up period was much longer, and for the long-term efficacy at last follow-up visit, both tendoscopic and open techniques provided satisfactory results, and there was no significantly difference between two groups.

Slade [7] firstly described the tendoscopic technique for de Quervain’s disease and reported satisfactory results with no significant complications. However, Kang et al. [10] reported that 3 (11%) patients in tendoscopic release group were involved with superficial radial nerve injury and that value in open release group was 9 (36%). Scheller et al. presented successful results with no nerve injury of operative treatment for de Quervain’s disease and Finkelstein’s test was negative in all 94 patients after a minimum of 15 years follow-up [19]. In our study, patients treated with tendoscopic release had lower rate of neurovascular injuries than open release: 1 (5%) in group A and 3 (14%) in group B (Table 2). No nerve injury was found in the tendoscopic release group. The only one case of cephalic vein injury occurred at the time of synovitis debridement, damaged by the shaver with excessive suction. The bleeding got relieved by manual compression finally. By contrast, 3 cases of radial sensory nerve injury occurred in the open release group but had no signs of neuroma. We started our tendoscopic technique since 2008. Of those 20 patients available for follow-up, the mean length of follow-up was 6.75 years (range, 4–10 years). Considering the anatomy of the fibro-osseous tunnel on the radial aspect, two transverse portals were made: one 1.5 cm distal and the other 2–2.5 cm proximal to the radial styloid. These two portals were alternatively used for visualization or manipulation, depending on the variations in the anatomy of the first dorsal compartment. Unlike two portals endoscopic technique, Karakaplan et al. [8] practiced one portal endoscopic release technique among 10 patients. In terms of complications, one patient had significant scar tenderness and two patients had paresthesia of the superficial radial nerve which was resolved spontaneously in 12 weeks postoperatively. In hand surgery, there is an increasing application of percutaneous needle technique for de Quervain’s disease; however, the outcomes were mixed. In a cadaver study by Güleç et al. [20], percutaneous needle release with an 18-G needle on 48 cadaver wrists was associated with high rates of incomplete release (47.9%) and tendon damage (39.6%) which was thought to be due to the presence of a septum. Lapègue et al. [21] applied ultrasound-guided percutaneous release of the first extensor tendon compartment with a 21-G needle; in this study, the clinical outcomes were feasible; however, the release was confirmed as “partial” in its cadaver study. By contrast, complete release under direct vision in our study was confirmed, which is essential to reduce postoperative recurrence of this disease.

Different with the outside-in technique of open procedure, tendoscopic technique provided inside-out release of the first extensor retinaculum, minimizing the rate of radial sensory nerve injury, as well as tendon injury and adhesion of subcutaneous tissue around the incisions. In our experience, synovial hyperplasia is with high prevalence in de Quervain’s disease, and synovial debridement could be well done under direct vision in tendoscopic technique. Meanwhile, by abducing the thumb, complete release of the retinaculum was confirmed with a 360° field view of the tendons under tendoscopy. In addition, our tendoscopic knife could be almost non-invasive for its retractable blade. The blade remained retracting inside of the blunt tip in the process of inserting and shuttling alongside the tendons.

**Fig. 5:**
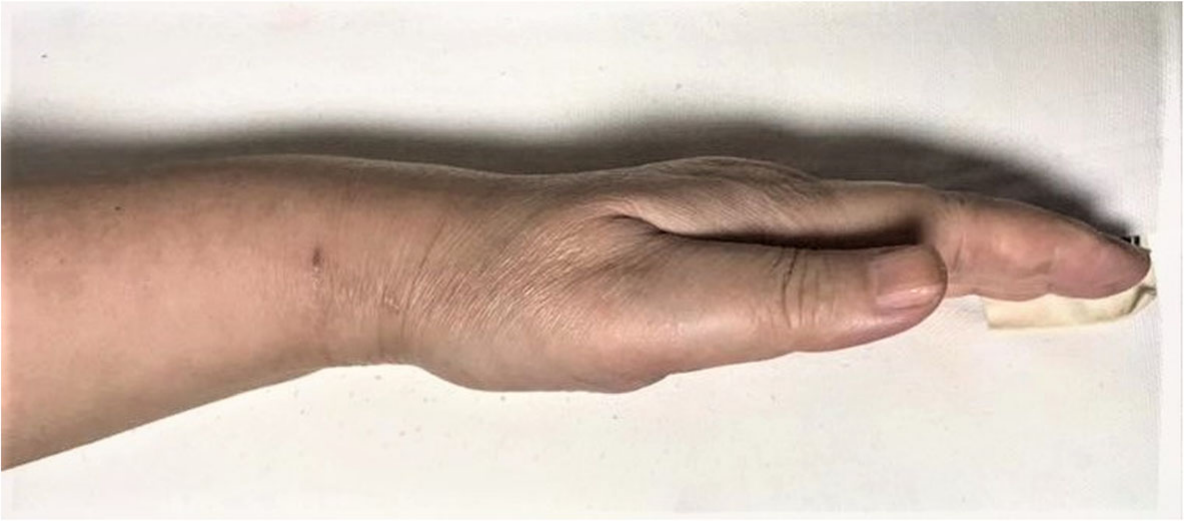
A 46-year-old female who underwent endoscopic release was satisfied with the scar in her wrist

Pregnant and perimenopausal women are more vulnerable to de Quervain’s disease, and esthetic appearance of the scar is really a considerable problem for this group that wrists are always uncovered by clothing. In our study, the POSAS score was applied for esthetic evaluation of the scar, both the patient’s subjective opinion (PSAS) and observer’s assessment (OSAS) showed that small transverse incisions in tendoscopic release group (Fig. 5) are with higher satisfaction than those longer longitudinal incisions in open release group, which exactly meets the medical esthetics. As a minimally invasive technique, tendoscopic release offers good visualization of the involved structures and equivalent decompression without the need for an extensive and more aggressive open approach. However, the manipulable space of wrist arthroscopy is much narrower than the arthroscopy of the knee and shoulder. In addition, the tendoscope and the other devices are nearly on the same axial line; this face-to-face approach has a steep learning curve and demands some arthroscopic skills.

In our experience,the tendoscopic release for De Quervain's Disease must pay attention to following several points. Understanding the surgical anatomy of the first extensor compartment and the adjacent structures is essential. Marking of the cephalic vein and the radial sensory nerve before surgery could be helpful. Sharp dissection through the subcutaneous tissue is avoided. The operator should use the shaver carefully with the blade back to the neurovascular structures and the vacuum suction should be opened after partial synovial debridement to avoid suction-induced neurovascular injury. Finally, professional arthroscopic instruments (e.g., the telescopic knife and the 3.0-mm shaver) would be favorable to the enhancement of operative efficiency and effect.

## Conclusions

The long-term results of our study are encouraging and demonstrate that tendoscopic release technique for de Quervain’s disease could provide earlier symptom relief and earlier recovery with fewer complications and a more desirable scar, as well as equivalent successful long-term outcome when compared with traditional open release.

## Data Availability

The datasets generated during the current study are not publically available due to ethical restrictions but are available from the corresponding author on reasonable request.

## Abbreviations

APL: The abductor pollicis longus tendon
DASH: The Disabilities of the Arm, Shoulder and Hand
EPB: The extensor pollicis brevis tendon
OSAS: The Observer Scar Assessment Scale
POSAS: The Patient and Observer Scar Assessment Scale
PSAS: The Patient Scar Assessment Scale
VAS: Visual analog scale
